# Cross-Sectional Multicenter Biomonitoring Study on Genotoxicity and Oxidative DNA Damage in Oncology Healthcare Workers from Seven Italian Hospitals

**DOI:** 10.3390/jox16010012

**Published:** 2026-01-13

**Authors:** Cinzia Lucia Ursini, Giorgia Di Gennaro, Giuliana Buresti, Raffaele Maiello, Anna Maria Fresegna, Aureliano Ciervo, Marco Gentile, Virginia Di Basilio, Sabrina Beltramini, Daniela Gaggero, Nicoletta Rigamonti, Erica Maccari, Giorgia Zorzetto, Piera Maiolino, Pasquale Di Filippo, Maria Concetta Bilancio, Paolo Baldo, Valeria Martinello, Andrea Di Mattia, Chiara Esposito, Patrizia Nardulli, Mariarita Laforgia, Maria Vittoria Visconti, Matteo Vitali, Emanuela Omodeo-Salè, Delia Cavallo

**Affiliations:** 1 Department of Occupational and Environmental Medicine, Epidemiology and Hygiene, Italian Workers’ Compensation Authority—INAIL, Monte Porzio Catone, 00078 Rome, Italyd.cavallo@inail.it (D.C.); 2 Department of Public Health and Infectious Diseases, University of Rome “La Sapienza”, 00185 Rome, Italy; 3 Pharmacy Complex Unit, IRCCS Ospedale Policlinico San Martino, 16132 Genoa, Italy; 4 Department of Pharmacy, Veneto Institute of Oncology IOV IRCCS, 35128 Padua, Italy; 5 Hospital Pharmacy, National Cancer Institute IRCCS Giovanni Pascale, 80131 Naples, Italy; 6 Hospital Pharmacy Unit, Centro Riferimento Oncologico CRO-Aviano—IRCCS, National Cancer Institute Aviano, 33081 Aviano, Italy; 7 Pharmacy Unit, Campus Bio-Medico University Hospital Foundation, 00128 Rome, Italy; 8 Pharmacy Unit, National Cancer Research Centre Giovanni Paolo II, 70124 Bari, Italy; 9 Hospital Pharmacy Department, European Institute of Oncology IRCCS, 20141 Milan, Italy

**Keywords:** antineoplastic drugs, human biomonitoring, biomarker, fpg-comet assay, occupational exposure

## Abstract

Cancer cases have been estimated that will increase in the next years with consequent increase of antineoplastic (AD) drug treatments and workers handling these hazardous chemicals. We aimed to evaluate genotoxic/oxidative effects of AD exposure by fpg-comet assay on a large size sample of workers (214 exposed and 164 controls) involved in preparation; administration, including Hyperthermic intraperitoneal chemotherapy (HIPEC) and pressurized intraperitoneal aerosol chemotherapy (PIPAC); and disposal. With the final aim to identify suitable early biomarkers of genotoxic effect useful to health surveillance, we correlated fpg-comet assay (blood) and Buccal Micronucleus Cytome (BMCyt) assay data. Fpg-comet parameters resulted higher in the exposed group vs. controls, demonstrating direct and oxidative DNA damage in workers handling ADs. Fpg-comet direct DNA damage and genotoxic parameters of BMCyt assay demonstrated a weak statistically significant correlation. This cross-sectional study is one of the few available evaluating both direct and oxidative DNA damage due to ADs on a large sample size of workers and correlating fpg-comet and BMCyt assay results. It highlights the need to evaluate genotoxic effects by both the biomarkers and furnishes a contribution to their validation. Moreover, we demonstrate for the first time oxidative DNA damage on workers performing HIPEC and PIPAC administration.

## 1. Introduction

It has been estimated that the number of new cancer cases will increase worldwide from 2022 to 2045 from 20 million to 32.6 million, as reported on the interactive web-based platform presenting global cancer statistics (Global Cancer Observatory) by IARC during 2024, consequently the total of anticancer drugs will increase together with the number of workers handling them.

Several antineoplastic drugs (ADs) meet the criteria for classification as carcinogen (categories 1A or 1B), mutagenic (categories 1A or 1B) or reprotoxic (categories 1A or 1B) in accordance with Regulation (EC) No 1272/2008, therefore they are included among the hazardous medical products falling under the scope of Directive 2004/37/EC.

Numerous ADs are included also in the document “NIOSH List of Hazardous Drugs in Healthcare Settings, 2024” that updated the previous lists published starting in 2010 replacing them. The Table 1 of this document includes drugs classified by the National Toxicology Program (NTP) [1] as “known to be a human carcinogen” and those classified by the International Agency for Research on Cancer (IARC) as Group 1 “carcinogenic to humans” or Group 2A “probably carcinogenic to humans”. In addition, also drugs identified as IARC Group 2B “possibly carcinogenic to humans” or as NTP “reasonably anticipated to be a human carcinogen” are included in Table 1 because they have manufacturer’s special handling information (MSHI).

Comet assay represents a widely used biomarker of early genotoxic effect in biomonitoring studies including those on healthcare sector [2]. In fact, this versatile method has been applied on a lot of studies on genotoxic effects of antineoplastic drugs, as also reported by Gianfredi et al. 2020 [3] and Ladeira et al. 2024 [2]. The Gianfredi’s systematic review and meta-analysis found a statistically significant association between occupational exposure to antineoplastic drugs and DNA damage evaluated by comet assay [3]. In their 2024 scoping review on comet assay in biomonitoring studies, Ladeira et al. analyzed 18 observational studies on healthcare workers exposed to antineoplastic drugs. They found that 68.4% of these studies found a statistically significant increase in DNA damage among exposed workers compared to controls [2].

However, currently, only two studies were performed on large size samples: the study of Huang et al. 2022 evaluating 305 exposed and 150 unexposed subjects in China [4] and the study of Sasaki et al. 2008 on 121 exposed workers and 46 controls in Japan [5]. Both the studies evaluated direct DNA damage and found statistically significant differences between exposed and unexposed subjects with higher values of different comet parameters in the exposed group. Huang et al. 2022 [4] conducted a meta-analysis to assess the relationship between occupational exposure to antineoplastic drugs and cytogenetic damage among healthcare workers. Their findings demonstrated a statistically significant association between such exposure and the occurrence of cytogenetic damage [6]. The comet assay method is a widely used method applied in biomonitoring studies of workers exposed to chemicals [2]. In particular, the alkaline comet assay is able to detect single- and double-strand DNA breaks, alkali-labile lesions converted to strand breaks under alkaline conditions, and single-strand breaks associated with incomplete excision repair [7]. It is to be highlighted that this method allows to evaluate also oxidative DNA damage using formamido-pyrimidin glycosylase (fpg) that recognizes and cuts the oxidized bases [8]. So, this method allows the simultaneous detection of the single- and double-strand DNA breaks (direct DNA damage) and the oxidized DNA bases (oxidative DNA damage) in the same sample. However, most of the available studies on genotoxic effects of ADs furnished only results of direct DNA damage and some of them showed lack of DNA damage evaluated by comet assay.

Most of ADs induce increase of Reactive Oxygen Species (ROS) production in cancer cells and this can induce oxidative DNA damage. In particular, the main drugs that increase ROS in cancer cells are doxorubicin, epirubicin, daunorubicin, alkylating agents, cisplatin, carboplatin, oxaliplatin, topotecan and irinotecan [9]. The involvement of ROS in the mechanism of action of ADs has been demonstrated [10,11].

This study is part of a larger project involving a Network of oncological hospitals and evaluating workplace contamination, cyto-genotoxic effects on buccal cells by Buccal Micronucleus Cytome assay (BMCyt) assay (whose results, expressed as mean frequency values of nuclear and cellular anomalies were reported in Ursini et al. 2025 [12]), and genotoxic/oxidative damage by fpg-comet assay. BMCyt assay represents a very interesting no-invasive biomarker of early effect, able to measure early cyto-genotoxic effects of exposure to genotoxic agents, since it allows the detection of Micronucleus (extranuclear fragments of acentric chromatid/chromosome fragments or whole chromatids/chromosomes that lag behind at the anaphase of dividing cells [13] and other cellular anomalies associated with chromosomal instability such as Nuclear Buds [14]).

In the present cross-sectional multicentre biomonitoring study the aims were:

i. to evaluate direct and oxidative DNA damage by fpg-comet assay in a large sample of healthcare workers handling ADs, including those involved in antineoplastic drug (AD) preparation, administration (in day hospital/ward and, for the first time, in the operating room via HIPEC—Hyperthermic Intraperitoneal Chemotherapy—and PIPAC—Pressurized Intraperitoneal Aerosol Chemotherapy), and disposers, also with the goal of identifying which tasks are at higher risk for genotoxic/oxidative effects; ii. to compare and correlate the parameters of the fpg-comet assay with those obtained previously in the same populations by the Buccal Micronucleus Cytome (BMCyt) assay, in order to identify suitable and well-accepted early biomarkers of genotoxic effect; iii. to analyze data of workplace and personal contamination obtained by wipe tests and pads from each hospital.

## 2. Materials and Methods

### 2.1. Subjects

An Italian Network of seven Italian hospitals was established to enrol workers handling mixtures of ADs and controls. The potentially exposed subjects (n = 213) were recruited among workers involved in the AD administration (including workers of operating rooms by performing Hyperthermic intraperitoneal chemotherapy (HIPEC) procedure and pressurized intraperitoneal aerosol chemotherapy (PIPAC)), preparation and cleaning. Preparators, who may be laboratory technicians, nurses, or pharmacists, are responsible for preparing and diluting anticancer drugs. They ensure the correct handling and distribution of these drugs. In some cases, especially in sterile laboratory settings, they also manage the disposal of drug residues. Administrators, typically nurses, they manage drug infusions in controlled environments using closed systems and ensure the safe disposal of unused drugs. Disposal, Social-Healthcare Operators (OSS) and auxiliary staff handle healthcare waste, including residual anticancer drugs, in compliance with regulations. They support patients in daily activities, maintain cleanliness and hygiene in hospital environments, assist healthcare personnel, transport materials, and ensure the proper disposal of healthcare and biological waste. Operating Room Staff, Physicians, nurses, and perfusionists involved in HIPEC and PIPAC procedures administer pressurized or heated chemotherapy during surgery. They monitor patients for adverse reactions, manage and safely dispose of contaminated waste and instruments, and work closely with the surgical team to maintain safety standards for both patients and staff, using appropriate personal protective equipment (PPE). Information regarding age, gender, smoking habits and job seniority were obtained by a questionnaire. A total of 165 workers non handling antineoplastic drugs were recruited in the same hospitals and considered as control subjects. The participants were recruited by the staff of oncology pharmacies who explained the aims of the study and the experimental procedures. Workers who participated in this study were not rewarded. All enrolled workers gave their informed consent before their inclusion in the study and their privacy rights have been observed. The Ethical Committee of the Oncological Unit coordinating the network approved the study (23 February 2022 approval n. R1624-22IEO 1735) that has been performed in accordance with the ethical standards laid down in the 1964 Declaration of Helsinki (https://www.wma.net/policies-post/wma-declaration-of-helsinki/, accessed on 10 January 2026) and its later amendments. During the same experimental campaign, we collected blood samples for comet assay and Buccal cells to perform Buccal Micronucleus Cytome (BMCyt) assay whose results (obtained on 200 exposed subjects and 150 controls with at least 2000 cells and expressed as mean values of all the detected abnormalities) have been previously published [12]. BMCyt assay method was described by Ursini et al. 2025 [12], briefly, oral exfoliated buccal cells were collected with a wet toothbrush by scraping the right and left cheeks, washed, fixed, stained with acridine orange, and analyzed by fluorescence microscope to detect cells with micronucleus (MN) nuclear buds (NB), broken eggs (BE), binucleated cells (BIN), karyolytic cells and cells with condensed chromatin (CC). The frequencies of cells with cellular or nuclear abnormalities were calculated on almost 2000 scored cells and expressed as ‰.

### 2.2. Analysis of Workplace and Personal Monitoring Data

Workplace and personal monitoring of Gemcitabine (GEM), Ifosfamide (IFO), Cyclofosfamide (CP), 5-Fluorouracil (5-FU) and Pt compounds (Pt) were carried out to detect AD contamination whose sampling methods and measurements were already described and published in Ursini et al. 2025 [12]. Potential skin exposure was measured using three pads worn on the forearms and thorax of each exposed worker during the workday according to Sottani et al. 2022 method [15]. All the Units used the same sampling protocol, and the analysis was conducted (by the Environmental Research Center, Istituti Clinici Scientifici Maugeri IRCCS, of Pavia) using high-performance liquid chromatographic method coupled with a tandem mass spectrometer (UHPLC MS/MS, Agilent Technologies, Lexington, CA, USA), according to Sottani et al. 2022 method [15] and by ICP-MS (Perkin Elmer, Shelton, CT, USA) to analyze Pt compounds. The LOD for Gemcitabine, Ifosfamide and Cyclofosfamide is 0.1 ng, whereas the LOD for 5-FU and Pt compounds are 5.0 and 0.008 ng respectively. In the present study, we conducted a detailed analysis of wipe and pad data collected from the workplaces where antineoplastic drug preparation and administration took place in each participating hospital.

### 2.3. Direct/Oxidative DNA Damage—Fpg Comet Assay

At start-shift of the third working day, whole venous blood samples from exposed and controls were collected by specialized medical personnel by venipuncture in sterile heparinized disposable syringes and transferred at 4 °C, within the same day, to the laboratory where they were frozen at −80 °C and then used to evaluate direct and oxidative DNA damage within one year from the collection.

We used Comet assay modified with the enzyme Fpg, a glycosylase (formamidopyrimidine DNA glycosylase), which recognizes and specifically cuts the oxidized bases (principally 8-oxoguanine) from DNA, producing apurinic sites converted in breaks by the associated AP-endonuclease activity that detected by comet assay as Fpg sites estimating oxidative DNA damage.

Frozen whole blood samples were used to perform fpg-comet assay. We followed the procedure of Collins et al. (1993) [8], with minor modifications [16]. We randomly selected images of 100 comets that were acquired and analyzed from each sample (either Fpg-treated or enzyme-untreated) with the image analyzer software (IAS) version 10.0 (Delta Sistemi, Roma, Italy). For each subject we calculated the mean values of comet parameters that indirectly measure DNA damage: tail DNA%, tail length (TL) and tail moment (TM). Tail DNA% represents the ratio of the tail intensity and total intensity of the comet measuring the number of broken pieces of DNA; comet TL detects the smallest detectable size of migrating DNA; TM is calculated as the product of TL and the tail DNA% providing an integrated measure that reflects both parameters. The combined use of these three parameters enables the estimation of a genotoxic agent’s capacity to fragment DNA strands into smaller or larger pieces. To assess direct DNA damage, for each subject we considered the mean values of Tail DNA%, TM, and TL obtained from enzyme-untreated cells. To evaluate oxidative DNA damage, we followed Collins et al. 2014 suggestions [17] using tail DNA % parameter, which provides the best estimate of the frequency of DNA breaks included those due to Fpg enzyme (relative to oxidized DNA bases). To obtain oxidative DNA damage (Fpg sites), we used tail DNA % from Fpg-enzyme treated cells (tail DNAenz%), that evaluates total (direct and oxidative) DNA damage, and we deducted tail DNA% from the tail DNAenz%. Individuals were classified as positive for oxidative DNA damage when the mean difference between tail DNAenz% and tail DNA% exceeded the established cutoff value of 4, in accordance with the criteria described by Cavallo et al. (2018) [18]. In addition, for each subject, we evaluated on 1000 cells of the Fpg-untreated sample, the percentage of comets (% comets) and the percentage of apoptotic cells (% apoptotic cells). Apoptotic cells were identified as those with a very small comet head and the majority of DNA located in the tail.

### 2.4. Statistical Methods

Statistical analyses were conducted using IBM SPSS Statistics for Windows, Version 25.0 (Armonk, NY, USA). The chi-square test and Fisher’s exact test were applied to evaluate the significance of associations between categorical variables and the groups under analysis. To assess differences in mean values between exposed and control subgroups, non-parametric tests were used, specifically the Mann–Whitney U test and the Kruskal–Wallis test. For pairwise comparisons, Dunn’s procedure with a Bonferroni correction for multiple comparisons was employed.

Multiple regression analyses were carried out, with the investigated effect biomarkers as dependent variables, and exposure along with potential confounders, such as age, gender, and smoking status as independent variables. A *p*-value < 0.05 was considered indicative of statistical significance.

A logistic regression model was also applied to evaluate the effect of exposure to antineoplastic drugs, adjusting for age, gender, and smoking habits. The outcome variable was oxidative damage, classified as either positive or negative (cutoff = 4). The model allowed us to estimate the odds ratios (ORs) and their corresponding 95% confidence intervals (95% CIs) for each independent variable, enabling the identification of significant risk factors associated with a positive oxidative damage outcome.

The Pearson correlation coefficient was also used to evaluate the possible correlation of DNA damage indicators with BMC Assay in the whole sample and in both the control and the exposed group.

## 3. Results

### 3.1. Study Population

Table 1A shows the characteristics of the studied population including those of each hospital. There were not statistically significant differences between exposed subjects and controls related to gender, smoking habit, age and job seniority. However, the analysis performed among workers included in each different task, showed differences for gender since the percentage of females in preparators was lower than in the other groups. Age resulted lower in the group of operating room that was younger than the other groups. All exposed subjects used disposable gowns, gloves and masks.

Table 1B shows that lifestyle factors such as alcohol consumption and dietary habits (fruits, fresh vegetables and grilled food intake), are similar in the two compared groups (exposed vs. controls).

### 3.2. Workplace and Personal Monitoring (Data Analysis)

Unlike our previous study [12], where the percentages of positivity for wipes were derived from data collected in the administration and preparation areas across all participating hospitals, resulting in a pooled analysis that did not distinguish between individual hospital settings, in the present study, we determined, for each hospital, the percentages of positivity on wipes, as well as the minimum and the maximum values observed as reported in Table 2. Notably, the percentage values pertaining to the operating room areas, which are exclusive to a single hospital, considered in the present article, have been reported previously as histograms [12]. Table 2 shows that the highest level of GEM was observed in an administration area (1162.91 ng/cm^2^), specifically on an armchair armrest, and in the pharmacy area (66.07 ng/cm^2^) on the APOTECA robotic system rotor. In the same hospital, were also recorded the highest CP concentrations, both in the administration area (143.51 ng/cm^2^, on the floor in front of an armchair) and in the preparation area (127.10 ng/cm^2^, on a clamp and an armrest). In addition, in the same hospital we found the highest Pt compounds maximum value in both administration area and the pharmacy, although with the lowest percentage of positive samples (59.6%). The highest level of IFO was measured in a pharmacy, reaching 9.63 ng/cm^2^ on the inside door handle in the preparation area. In contrast, among the administration areas, the highest IFO concentration resulted 0.84 ng/cm^2^. For wipes contaminated with 5FU, the highest concentration was detected in a pharmacy (49.69 ng/cm^2^), while the maximum value found among the administration areas was 31.93 ng/cm^2^. Platinum compounds reached their highest levels of 59.71 ng/cm^2^ on a transport container in an administration area, and 1973.93 ng/cm^2^ on a clamp and armrest in the pharmacy area.

Table 3 reports minimum and maximum values of drug concentrations observed across all pads worn by the workers in correspondence with left and right forearms and the thorax and the percentages of all monitored workers with at least one positive pad (Positives). Notably, we detected the highest concentration of GEM (52.85 ng/cm^2^) on a pharmacy worker and the highest CP level (10.42 ng/cm^2^) on a worker of the operating room of the same hospital. The highest level of IFO was found on a pharmacy worker (29.0 ng/cm^2^). The highest concentrations of 5FU (137.38 ng/cm^2^) and Platinum compounds (7.20 ng/cm^2^) were observed on administration staff.

### 3.3. Fpg-Comet Assay (Direct and Oxidative DNA Damage)

**Figure 1:**
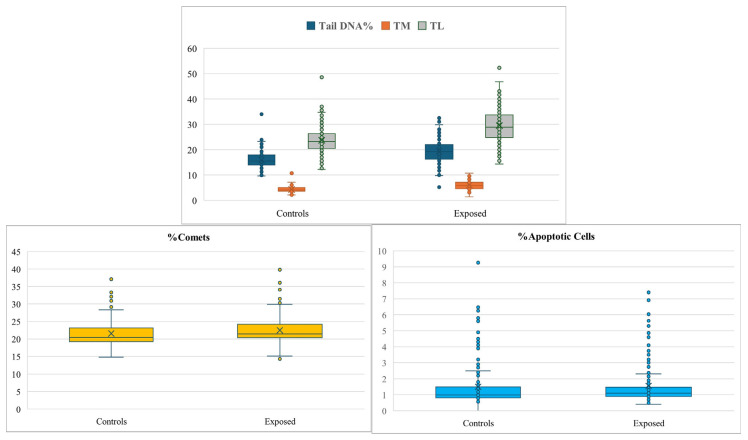
Representation of parameters indicating direct DNA damage and apoptosis. TM (Tail Moment) and TL (tail length).

Table 4 shows the results of fpg-comet assay reporting the analyzed parameters indicating direct DNA damage. It demonstrates that for the group of the exposed subjects, we found higher mean values of all the detected parameters compared with the control group. When we analyzed the different tasks, we found that both Tail DNA % and TL were higher in administrators and preparators compared to the control group, with no significant difference between administrators and preparators themselves. The TM parameter showed significant differences only between administrators and preparators, but not when compared to controls. The % Comet value was higher in exposed workers, especially among administrators and preparators, than in controls. The apoptotic cells were slightly higher (although the difference resulted statistically significant) only considering all the exposed group compared to controls. In Figure 1 the box-plots of direct DNA damage parameters, % comet and % apoptotic cells are reported and they show the mean values and the variability.

**Figure 2:**
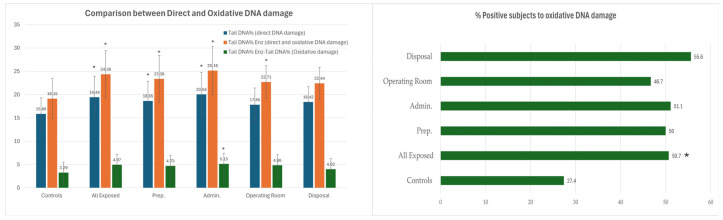
Fpg-comet assay results related to Oxidative DNA damage. Prep.: Preparators; Admin.: Administrators; OR: Operating Room performing HIPEC and PIPAC. TM: Tail moment; TL: Tail Length. * *p*-Value ≤ 0.001 (χ^2^ test) versus controls. Positive subjects (Subjects with *tail DNA%* enz-*tail DNA%* ≥ 4).

We found that oxidative DNA damage (Figure 2, first panel) was higher in the exposed subjects either in terms of difference (*tail DNA%* enz- *tail DNA%)* or in terms of percentages of subjects positive to oxidative DNA damage (Figure 2, second panel) with higher values in the exposed group (50.7% vs. 27.4%).

Relatively to the oxidative DNA damage for each task (Figure 2), the tail DNA% enz mean value (indicating total direct and oxidative DNA damage) was higher in administrators (including the group of the operating room performing HIPEC and PIPAC) while the oxidative damage parameter resulted higher in a way statistically significant only in the group of the administrators but not in the operating rooms and in the other groups. Whereas, the percentages of subjects positive to oxidative DNA damage were all higher with values ranging from 46.7% (operating room) and 55.6% (disposal) than in the controls (27.4%) (Figure 2, second panel).

Table 5 presents the results of the multiple regression model used to estimate the effect of exposure to ADs on biomarker outcomes, while adjusting for potential confounders such as age, gender, and smoking habits. The analysis showed a statistically significant effect of AD exposure on the following biomarkers: Tail DNA %, Tail DNA% Enz, Oxidative Damage, Tail Moment and Tail Length. Specifically, exposed workers showed increases of 3.59%, 5.28%, 1.69%, 1.79%, and 5.75%, respectively, in these biomarkers compared to non-exposed workers, with all associations reaching statistical significance (*p* < 0.05).

Smoking habits were negatively associated with % Comets (*p* = 0.007) and % apoptotic cells (*p* < 0.001) although with very low decrease of 0.72 and 0.36 respectively. Age did not emerge as a significant predictor for most biomarkers, except for %Comets, where it showed a positive and statistically significant association (*p* = 0.035), suggesting that increasing age is related to a higher percentage of damaged cells (with different levels of DNA damage) as measured by the comet assay.

A logistic regression model was also applied to evaluate the effect of exposure to ADs on oxidative damage positivity outcome, adjusting for age, gender, and smoking habits (Table 6). The outcome variable oxidative damage was classified either positive or negative (cut-off = 4). The model estimated odds ratios (ORs) and 95% confidence intervals (CIs) for each variable, identifying significant risk factors for positive oxidative damage.

Workers exposed to ADs are 2.6 times more likely to be positive for oxidative DNA damage compared to unexposed workers, after adjusting for age, gender, and smoking habits. This effect is statistically significant (*p* < 0.001). There are non-significant effects due to age, gender and smoking habits.

Table 7 presents the correlation between age and DNA damage biomarkers, showing no significant association between age and the biomarker outcomes.

### 3.4. Association Between Fpg-Comet and BMCyt Assay

With the aim to evaluate the overall impact of AD exposure, we investigated the possible correlation between direct/oxidative DNA damage parameters obtained by comet assay and the frequency of cells with micronucleus (MN), nuclear buds (NB) or broken eggs (BE) (indicative of genotoxicity) and the frequency of the other BMCyt assay parameters detected on the same subjects whose buccal cells were collected simultaneously to blood and published in Ursini et al. 2025 [12] where we reported the mean frequencies of the analysed parameters.

**Figure 3:**
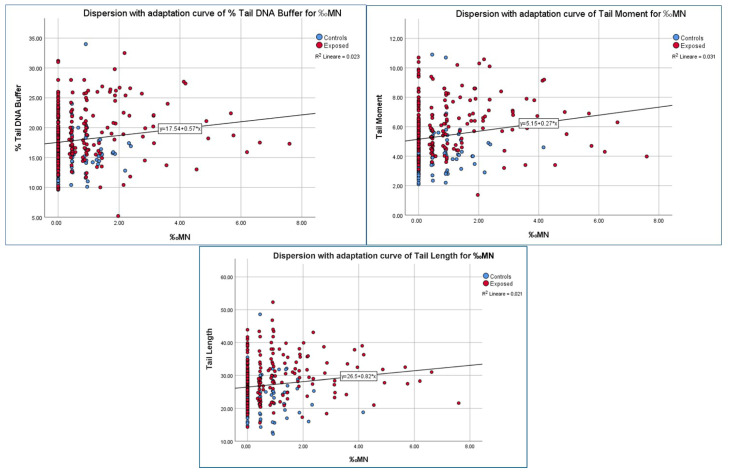
Correlation between Micronucleus Frequency and Comet Assay Parameters in Control and Exposed Groups.

Table 8 shows Pearson correlation coefficients (r) and corresponding *p*-values (*p*) across the total sample, controls, and exposed group and Figure 3 shows the simple dispersions with the adaptation curves relative to the variables resulting correlated between direct DNA damage parameters and frequency of micronucleated cells.

As showed in Table 8, some correlations are statistically significant in the total sample, such as between “‰MN—%DNA tail Buffer” (r = 0.151, *p* = 0.005), “‰MN—Tail Moment” (r = 0.177, *p* = 0.001) and tail length (r = 0.146, *p* = 0.007), suggesting a weak association between micronucleus frequency and direct DNA damage parameters. Table 8 also shows that in both the Control and Exposed groups, correlations are generally weaker and not statistically significant.

## 4. Discussion

This cross-sectional biomonitoring study evaluated direct and oxidative DNA damage by the very sensitive fpg-comet assay on blood of a large size worker population handling ADs and demonstrated that, to date, we find genotoxic and oxidative effects due to AD exposure. We also demonstrated ADs contamination in all the workplaces furnishing the ranges of concentrations found on wipes and pads and these data are indicative of a potential source of exposure.

In our previous study [12], we showed the percentages of wipes and pads with detectable value of ADs concentrations where Gemcitabine, Pt compounds and 5-FU resulted on wipes the drugs present in the highest percentage. In particular, in the pharmacy, Pt compounds and 5-FU resulted the drugs with the highest percentages of contaminated wipes, whereas the administration area resulted with the highest percentages of wipes contaminated with GEM and Pt compounds. Pt compounds and 5-FU resulted the drugs with the highest percentages of contaminated pads in both pharmacy and administration areas.

In the present study, where we calculated from the same data the ranges of concentrations found in each detected hospital, we show that the highest concentrations of 5-FU and Pt compounds were on wipes collected in the pharmacy, the highest concentration of GEM was found in administration area, whereas CP resulted with higher maximum values in administration in respect to pharmacy in one hospital with values very higher than those found in the other hospitals.

In addition to workplace surface contamination detection and personal monitoring using pads, Ursini et al. 2025 [12] also investigated the presence of ADs in the urine of the same exposed subjects whose blood samples were collected for the Fpg-comet assay. No detectable levels of the analyzed drugs were found in the urine of these workers demonstrating a very low occupational exposure. Therefore, the statistically significant direct and oxidative DNA damage observed in the present study in exposed workers compared to controls (matched for confounding factors such as age, smoking habits, alcohol consumption, fruit and vegetable intake, and grilled food consumption), confirms that the comet assay is a highly sensitive biomarker for detecting potential early genotoxic effects in workers handling antineoplastic drugs (ADs).

Relatively to the identification of the tasks at higher risk, we found that both preparation and administration tasks could induce genotoxic effects.

Our study represents one of the few available ones that evaluated also oxidative DNA damage by fpg-comet assay on workers exposed to ADs demonstrating that exposure to these drugs could induce oxidative DNA damage on blood of workers handling ADs. All the groups of exposed workers, including workers administering ADs in the operating rooms by HIPEC and PIPAC procedures, showed induction of oxidative DNA damage in terms of percentage of positive subjects. The results relative to operating room administrators, at the best of our knowledge, are available for the first time.

In Ursini et al. 2025, BMCyt assay, performed on the same populations of exposed and controls, found on buccal cells higher mean values of all genotoxicity parameters including cells with micronucleus and, analysing the tasks, it showed that both preparation and administration are able to induce early genotoxic effects [12]. Workers performing HIPEC and PIPAC in operating rooms showed higher mean frequency values of MN + NB + BE than in the control group indicating genotoxicity in respect to controls. Therefore, both buccal cells and blood represent good biological matrices where to detect potential early genotoxic effects on workers administering ADs by HIPEC and PIPAC. We also found detectable levels of drug contamination in the wipes collected in the operating rooms with 5-FU positive in the 43.7% of samples reaching the highest concentration of 5.24 ng/cm^2^ and detectable value of CP also on pads.

Also, Ndaw et al. 2018 detected Platinum in operating rooms during HIPEC and PIPAC procedures and they found significant workplace contamination [19]. Cisplatin and doxorubicin contamination were found on the operating room surfaces even after a cleaning protocol [20]. Also, Delafoy et al. 2023 found AD contamination in the operating room highlighting the need to improve training programs for all the workers handling ADs including those of operating rooms [21].

Our present study confirms the results of a lot of available studies performed by comet assay on worker populations less numerous than our population as reported in the review of Ladeira et al. 2024 [2], and are also in agreement with those obtained by Huang et al. 2022 on 305 exposed workers (nurses handling ADs) and 150 controls (healthy nursing staff members not handling these drugs) that demonstrated positive relation between exposure to ADs and risk of DNA damage evaluated by the comet and cytokinesis-block micronucleus assays in nurses [4]. Rekhadevi et al. 2007, that performed comet assay and MN assay on both lymphocytes and buccal cells from 60 nurses of an Indian hospital (exposed workers) and 60 controls, found statistically significant differences between exposed and controls with higher mean values of comet tail length and MN frequency in the exposed group [22].

Unlike previous listed studies that reported statistically significant genotoxic and oxidative effects in exposed healthcare workers, other studies—including our previous [23] study and another Italian research [24]—did not show statistically significant differences between exposed and control groups [23,24,25,26]. In Ursini et al. 2006 [23] TM was the only detected comet parameter, whereas in the present study we detected also tail DNA% which provides the best estimate of the frequency of DNA breaks. The other Italian study [24] did not find significant differences between controls and exposed workers, and the Authors explained these results with the stringent application of the guidelines published in Italy to prevent ADs occupational exposure and a crosslinking effect. This suggests that, under certain conditions such as specific comet parameters, sample size and strict safety protocols, handling antineoplastic drugs not always result in detectable DNA damage.

Regarding oxidative DNA damage induction, some studies [27,28,29,30] consistently show that nurses handling antineoplastic drugs experience measurable changes in biomarkers of oxidative stress, such as increased catalase (CAT) activity, higher levels of thiobarbituric acid reactive substances (TBARS), and elevated malondialdehyde. These changes indicate that occupational exposure to these drugs can lead to increased oxidative stress and lipid peroxidation, therefore, the results observed in the present study related to oxidative DNA damage agree with the above cited studies.

Another study [31] performed in Brazil on 49 exposed subjects and 10 controls used both comet assay and Micronucleus assay on exfoliated buccal cells to evaluate genotoxic effects of AD exposure. They also found increased DNA damage and frequency of MN in the exposed subjects compared with the unexposed workers.

The present study found results in agreement with those obtained on buccal cells [12] relative to genotoxic effects of ADs exposure, but in addition it demonstrated oxidative DNA damage induction suggesting that both the biomarkers are useful to assess the early genotoxic and oxidative effects of ADs exposure.

Relatively to our evaluation of possible association between fpg-comet and BMCyt assay biomarkers we found a weak association although statistically significant between the assays confirming the results of Santos et al. 2020 [31] that found a positive correlation between the results of the two biomarkers of effect comet and BMCyt assays reporting a positive correlation between the frequency of genomic lesions (by comet assay) and frequency of permanent damage (by micronucleus assay). MN can be represented by acentric chromatid/chromosome fragments originated after extensive DNA damage such as Double Strand breaks if misrepaired, as suggested by Luzhna et al. 2013 [13], but MN can also be represented by whole chromatids or chromosomes caused by mitotic spindle failure, kinetochore damage, centromeric DNA hypomethylation, and defects in the cell cycle control system, as suggested by Mateuca et al. 2006 [32]. Therefore, this different nature of MN could explain the weak correlation between comet parameters indicating single and double DNA Strand breaks and cells with MN.

The detected genotoxic/oxidative effects found in this study and in all our project highlight the need to better inform and form the workers on the possible early genotoxic and oxidative effects of ADs handling with the final aim to to raise awareness and the perception of risk related to exposure to antineoplastic drugs. To minimize the risk of exposure as much as possible, workers must strictly follow available guidelines and correctly use personal protective equipment (PPE). Proper use of PPE also helps to reduce contamination of work surfaces and clothing.

These measures are essential to protect the health and safety of healthcare workers who handle hazardous drugs, and to maintain a safe working environment.

## 5. Conclusions

This research demonstrated workplace AD contamination and induction of early genotoxic and oxidative effects of ADs exposure on blood of workers handling these hazardous drugs. This study represents the only available one on the evaluation of genotoxic and oxidative effects induced on blood during administration in operating room (HIPEC and PIPaC) together with the other study performed by our laboratory on buccal cells conducting the BMCyt assay [12].

This cross-sectional multicentre biomonitoring study, involving the most important oncological Italian Institutes and performed on a large size sample, suggests the need to use both fpg-comet and BMCyt assays to evaluate early genotoxic and oxidative effects of exposure to mixtures of ADs representing useful tools to evaluate the risk of exposure to these drugs in healthcare workers.

The overall results obtained in this study have made it possible to highlight that the following actions are still needed:

- Better inform and form workers on the potential risk of these drug mixtures.
- Raise awareness and assess risk perception, ensuring that all staff understand the dangers associated with handling antineoplastic drugs and regularly checking how well they perceive these risks.
- Follow guidelines by adhering to official protocols and safety procedures designed to protect workers from hazardous drug exposure.
- Use PPE correctly to shield oneself from contact with dangerous substances.
- Minimize exposure and contamination by taking all necessary precautions and to prevent the spread of contamination to surfaces and clothing in the workplace.

## Figures and Tables

**Table 1:** (**A**). General characteristics of studied population. (**B**). Alcohol Consumption and Dietary Habits of studied population.

(**A**)									
**Sample**	**Gender**		**Smoking Habit**			**Age**		**Job Seniority**	
	**Males**	**Females**	**Yes**	**No**	**Former**	**Mean ± SD**	**[Range]**	**Mean (Years ± SD)**	**[Range]**
	**N (%)**	**N (%)**	**N (%)**	**N (%)**	**N (%)**				
Hospital A	11 (18.6)	48 (81.4)	6 (10.0)	44 (73.3)	10 (16.7)	41.24 ± 10.23	[23–65]	12.55 ± 9.56	[0.3–34]
Hospital B	4 (19.0)	17 (81.0)	4 (21.1)	14 (73.7)	1 (5.3)	36.57 ± 8.39	[25–53]	7.25 ± 8.20	[1–25]
Hospital C	12 (20.0)	48 (80.0)	7 (14.0)	41 (82.0)	2 (4.0)	46.68 ± 11.47	[26–65]	10.23 ± 9.29	[0.1–30]
Hospital D	20 (28.2)	51 (71.8)	3 (4.3)	66 (95.7)	0 (0.0)	44.00± 11.46	[22–63]	10.73 ± 8.90	[0.2–28]
Hospital E	19 (45.2)	23 (54.8)	18 (43.9)	18 (43.9)	5 (12.2)	41.78 ± 9.01	[26–65]	8.05 ± 5.41	[1–18]
Hospital F	16 (18.0)	73 (82.0)	11 (14.7)	55 (73.3)	9 (12.0)	41.08 ± 11.38	[25–66]	6.27 ± 7.47	[0.3–30]
Hospital G	12 (34.3)	23 (65.7)	8 (25.0)	17 (53.1)	7 (21.9)	40.26 ± 8.37	[26–66]	12.29 ± 7.11	[0.3–26]
Total (N = 378)	94 (24.9)	283 (75.1)	57 (16.5)	255 (73.7)	34 (9.8)	42.30 ± 10.82	[22–66]	9.29 ± 8.45	[0.1–34]
Controls (N = 164)	46 (28.0)	118 (72.0)	21 (13.9)	113 (74.8)	17 (11.3)	42.93 ± 10.63	[23–65]	7.30 ± 7.45	[0.3–30]
Exposed (N = 214)	48 (22.5)	165 (67.5)	36 (18.5)	142 (72.8)	17 (8.7)	41.82 ± 10.96	[22–66]	9.51 ± 8.54	[0.1–34]
Preparators (N = 58)	26 (44.8)	32 (55.2)	4 (7.4)	42 (77.8)	8 (14.8)	43.71 ± 11.54	[25–66]	9.10 ± 9.94	[0.1–28]
Administrators (N = 132)	17 (13.0)	114 (87.0)	27 (22.5)	85 (70.8)	8 (6.7)	41.57 ± 10.45	[22–66]	10.28 ± 8.86	[0.2–34]
Operating Room (N = 15)	4 (26.7)	11 (73.3)	3 (23.1)	9 (69.2)	1 (7.7)	32.67 ± 7.46	[25–50]	3.01 ± 1.51	[1–6]
Disposal (N = 9)	1 (11.1)	8 (88.9)	2 (25.0)	6 (75.0)	0 (0.0)	48.56 ± 11.28	[30–63]	11.11 ± 10.17	[1–30]
***p*-Value** (Exp vs. Contr)	0.220 ^a^		0.432 ^a^			0.298 ^c^		0.282 ^c^	
***p*-Value** (Different Tasks)	**0.001 ^a^**		0.173 ^b^			**0.002 ^d^**		0.065 ^d^	
						* OR vs. Prep/Adm/Disposal/Contr			
(**B**)									
	**Consumption**				**Total (N = 378)**	**Controls (N = 164)**	**Exposed (N = 214)**	***p*-Value**	
					**N (%)**	**N (%)**	**N (%)**		
Alcohol	None				167 (49.4)	75 (47.8)	92 (50.8)	0.833 ^a^	
	Spirits occasionally				61 (18.0)	27 (17.2)	34 (18.8)		
	Wine/beer				51 (15.1)	25 (15.9)	26 (14.4)		
	Wine/beer/spirits occasionally				59 (17.5)	30 (19.1)	29 (16.0)		
Fruit	Rarely/never				60 (17.3)	20 (12.6)	40 (21.4)	0.052 ^a^	
	Once a day				172 (49.7)	79 (49.7)	93 (49.7)		
	Several times a day				114 (32.9)	60 (37.7)	54 (28.9)		
Fresh Vegetables	Rarely				31 (9.0)	12 (7.5)	19 (10.2)	0.650 ^a^	
	Once a day				160 (46.2)	73 (45.9)	87 (46.5)		
	Several times a day				155 (44.8)	74 (46.5)	81 (43.3)		
Grilled foods	Never and rarely				41 (12.1)	18 (11.6)	23 (12.4)	0.716 ^a^	
	Once a month				122 (35.9)	53 (34.2)	69 (37.3)		
	2–3 times/month				134 (39.4)	61 (39.4)	73 (39.5)		
	More than 2–3 times/month				43 (12.6)	23 (14.8)	20 (10.8)		

OR: Operating Room. ^a^ χ2 test; ^b^ Fisher’s exact test; ^c^ Mann-Whitney Test; ^d^ Kruskal Wallis Test; * Dunn’s procedure with a Bonferroni correction for multiple comparisons. Operating Room vs. Controls (*p* = 0.004), vs. Preparators (*p* = 0.005); vs. Administrators (*p* = 0.023); vs. Disposal (*p* = 0.005). Bold character indicates statistically significant *p*-values.

**Table 2:** Percentage of wipes with detectable drug concentration (Positives) and Minimum and maximum concentrations.

	DEPARTMENT/AREA	DRUG	POSITIVES %	MIN (ng/cm^2^)	MAX (ng/cm^2^)
**HOSPITAL A**	Administration Ward	GEM	10.0	0.0070	0.0740
		IFO	16.7	0.0020	0.0440
		CP	16.6	0.0008	0.1125
		5-FU	66.6	0.0140	0.4700
		Pt	90.0	0.00001	0.0031
	Administration Day Hospital	GEM	46.7	0.0012	0.8823
		IFO	0	0	0
		CP	26.6	0.0036	0.0804
		5-FU	46.6	0.0149	0.0585
		Pt	96.6	0.00007	2.7188
	Pharmacy	GEM	38.5	0.002	0.4410
		IFO	15.4	0.072	0.7330
		CP	12.8	0.002	1.0660
		5-FU	51.3	0.013	32.8130
		Pt	100	0.00008	0.1254
**HOSPITAL B**	Administration Day Hospital	GEM	26.2	0.0008	0.13202
		IFO	26.2	0	0.4265
		CP	21.3	0	0.2760
		5-FU	50.8	0.0108	6.7928
		Pt	100	0.00002	0.1644
	Pharmacy	GEM	35.3	0.0040	1.9001
		IFO	20.6	0.0106	0.5403
		CP	19.1	0.0061	0.1626
		5-FU	47.1	0.0106	1.6575
		Pt	94.1	0.00001	0.0134
**HOSPITAL C**	Administration Day hospital	GEM	67.0	0.001	6.6555
		IFO	5.0	0.00108	0.1714
		CP	67.0	0.00048	2.8448
		5-FU	49.0	0.01400	8.1528
		Pt	100	0.000031	0.3117
	Pharmacy	GEM	22.0	0.0013	1.0398
		IFO	8.0	0.0013	1.3218
		CP	32.0	0.0003	0.3819
		5-FU	60.0	0.0017	5.3373
		Pt	100	-	0.06497 ^#^
**HOSPITAL D**	Administration Day hospital	GEM	100	0.0007	**1162.91**
		IFO	28.8	0.0008	0.1741
		CP	96.1	0.0003	**143.51**
		5-FU	92.3	0.0062	**31.93**
		Pt	100	0.0026	**59.71**
	Pharmacy	GEM	100	0.0123	**66.0681**
		IFO	61.5	0.0029	7.1297
		CP	96.1	0.0031	**127.1012**
		5-FU	75.0	0.0133	27.97
		Pt	59.6	0.00186	**1973.93**
**HOSPITAL E**	Administration Day hospital	GEM	98.2	0.0005	1.4366
		IFO	96.4	0.0005	0.1440
		CP	36.3	0.0002	0.0443
		5-FU	29.1	0.0157	0.67
		Pt	100	0.00011	0.1147
	Pharmacy	GEM	100	0.0011	1.5002
		IFO	100	0.0333	2.2638
		CP	100	0.0012	0.4130
		5-FU	83.0	0.0137	2.0019
		Pt	83.3	0.00235	0.033
**HOSPITAL F**	Operating room	GEM	6.25	-	0.0393 ^#^
		IFO	6.25	-	0.04320 ^#^
		CP	6.25	-	0.5157 ^#^
		5-FU	43.7	0.0006	5.2459
		Pt	100	0.00003	0.00061
	Administration Day Hospital	GEM	20	0.0017	4.317
		IFO	34.7	0.0024	0.4048
		CP	26	0.0021	0.6753
		5-FU	67.4	0.0015	4.0838
		Pt	100	0.00002	3.0997
	Pharmacy	GEM	20	0.0160	0.2493
		IFO	50	0.0020	**9.6327**
		CP	65	0.0024	1.8351
		5-FU	60	0.0033	1.8948
		Pt	100	0.00005	0.082
**HOSPITAL G**	Administration Day hospital	GEM	78.7	0.0007	4.5207
		IFO	15.3	0.002	**0.8416**
		CP	47.3	0.0003	1.5681
		5-FU	56.6	0.0140	2.9344
		Pt	100	0.0001	0.9227
	Pharmacy	GEM	64	0.015	2.202
		IFO	64.3	0.003	0.620
		CP	57	0.0014	0.9556
		5-FU	64	0.0142	**49.6858**
		Pt	100	0.0013	0.0193

^#^ The reported maximum corresponds to the only concentration measured above the LOD for this drug. Gemcitabine (GEM), Ifosfamide (IFO) and Cyclofosfamide (CP) LOD = 0.1 ng, 5-Fluorouracyl (5-FU) LOD = 5 ng and Pt compounds (Pt) LOD = 0.008 ng. Bold character indicates the highest value found in the specific detected workplace.

**Table 3:** Percentages of monitored workers with at least one positive pad and Minimum and maximum concentration.

	DRUG	POSITIVES %	MIN (ng/cm^2^)	MAX (ng/cm^2^)
**HOSPITAL A**	GEM	53.7	0.0080	1.0534
	IFO	25.0	0.0151	0.7277
	CP	21.8	0.0322	1.8061
	5-FU	46.9	0.0573	22.269
	Pt	100	0.0012	0.0268
**HOSPITAL B**	GEM	16.7	0.0984	0.1978
	IFO	16.7	0.5301	1.0787
	CP	16.7	0.2042	0.4521
	**5-FU**	33.3	0.0603	**137.3869**
	Pt	100	0.0001	0.0102
**HOSPITAL C**	GEM	28.0	0.0062	7.1449
	IFO	4.0	0.0297	21.1275
	CP	12.0	0.0073	0.5215
	5-FU	72	0.018	1.2431
	Pt	72	0.000037	0.00328
**HOSPITAL D**	GEM	20.6	0.0027	0.3100
	IFO	2.9	-	0.6510 ^#^
	CP	8.8	0.047	0.5667
	5-FU	47.0	0.0125	1.5563
	**Pt**	58.8	0.0101	**7.2086**
**HOSPITAL E**	GEM	57.1	0.0011	23.0000
	**IFO**	57.1	0.0017	**29.005**
	CP	52.4	0.0012	0.2745
	5-FU	52.4	0.0580	4.6829
	Pt	100	0.0001	0.0088
**HOSPITAL F**	**GEM**	39.2	0.0047	**52.8507**
	IFO	11.8	0.011	1.8634
	**CP**	15.7	0.7773	**10.4180**
	5-FU	35.3	0.0586	6.9453
	Pt	100	0.00016	0.2286
**HOSPITAL G**	GEM	30.0	0.0042	0.0927
	IFO	0	0	0
	CP	0	0	0
	5-FU	50.0	0.0117	2.0687
	Pt	33.3	0.0001	0.0222

^#^ The reported maximum corresponds to the only concentration measured above the LOD for this drug. Gemcitabine (GEM), Ifosfamide (IFO) and Cyclofosfamide (CP) LOD = 0.1 ng, 5-Fluorouracyl (5-FU) LOD = 5 ng and Pt compounds (Pt) LOD = 0.008 ng. Bold character indicates the highest value found for each drug.

**Table 4:** Fpg-comet assay results related to direct DNA damage.

	Tail DNA%	TM	TL	%Comets	%Apoptotic Cells
	Mean ± SD	Mean ± SD	Mean ± SD	Mean ± SD	Mean ± SD
Controls (N = 164)	15.88 ± 3.44	4.33 ± 1.28	23.72 ± 5.18	21.56 ± 3.83	1.51 ± 1.35
Exposed (N = 214)	19.44 ± 4.51	6.06 ± 1.78	29.51 ± 6.53	22.49 ± 3.66	1.56 ± 1.26
Prep. (N = 58)	18.65 ± 4.20	5.55 ± 1.67	28.70 ± 6.31	22.32 ± 3.09	1.28 ± 0.59
Admin. (N = 132)	20.04 ± 4.74	6.38 ± 1.80	30.36 ± 6.59	22.50 ± 3.92	1.74 ± 1.49
OR (N = 15)	17.85 ± 3.57	5.67 ± 1.47	27.54 ± 6.08	21.90 ± 2.44	1.00 ± 0.16
Disposal (N = 9)	18.42 ± 3.29	5.47 ± 1.88	25.67 ± 5.87	24.36 ± 4.63	1.69 ± 1.24
***p*-Value** (Exp vs. Contr)	**<0.001 ^c^**	**<0.001 ^c^**	**<0.001 ^c^**	**<0.001 ^c^**	**0.044 ^c^**
***p*-Value** (Different tasks)	**<0.001 ^d^**	**<0.001 ^d^**	**<0.001 ^d^**	**0.002 ^d^**	0.165 ^d^
	* Contr vs. Prep/Adm	* Prep vs. Adm	* Contr vs. Prep/Adm	* Contr vs. Prep/Adm	

Prep.: Preparators; Admin.: Administrators; OR: Operating Room performing HIPEC and PIPAC. TM: Tail moment; TL: Tail Length; ^c^ Mann-Whitney Test; ^d^ Kruskal Wallis Test; * Dunn’s procedure with a Bonferroni correction for multiple comparisons. Bold character indicates statistically significant *p*-values.

**Table 5:** Multiple regression model estimating the effect of antineoplastic drug exposure on biomarker outcomes, adjusting for confounders.

Biomarker	Independent Variables	Unstandardised B	95% CI		Standardised Beta	*p*-Value
			Lower	Upper		
Tail DNA%	Age	0.001	−0.040	0.041	0.001	0.979
	Gender ^a^	0.037	−0.956	1.031	0.004	0.941
	Smoking habits ^b^	0.541	−0.040	1.122	0.091	0.068
	**Exposure** ^c^	3.590	2.702	4.477	0.397	**<0.001**
Tail DNA% enz	Age	0.020	−0.027	0.067	0.040	0.406
	Gender	0.380	−0.763	1.523	0.031	0.513
	Smoking habits	0.510	−0.158	1.178	0.071	0.134
	**Exposure**	5.275	4.254	6.296	0.484	**<0.001**
Oxidative damage	Age	0.020	−0.015	0.055	0.060	0.256
	Gender	0.361	−0.483	1.205	0.045	0.401
	Smoking habits	−0.047	−0.541	0.446	−0.010	0.850
	**Exposure**	1.689	0.935	2.443	0.234	**<0.001**
Tail Moment	Age	0.004	−0.012	0.020	0.023	0.624
	Gender	−0.125	−0.509	0.259	−0.030	0.523
	Smoking habits	0.078	−0.147	0.302	0.032	0.497
	**Exposure**	1.790	1.447	2.133	0.489	**<0.001**
Tail Length	Age	0.011	−0.049	0.071	0.018	0.722
	Gender	−0.556	−2.019	0.908	−0.037	0.456
	Smoking habits	0.390	−0.466	1.246	0.044	0.371
	**Exposure**	5.749	4.442	7.057	0.427	**<0.001**
%Comets	**Age**	0.040	0.003	0.076	0.113	**0.035**
	Gender	−0.521	−1.414	0.371	−0.061	0.251
	**Smoking habits**	−0.719	−1.241	−0.197	−0.145	**0.007**
	Exposure	0.782	−0.015	1.579	0.103	0.055
%Apoptotic cells	Age	0.011	−0.002	0.024	0.086	0.108
	Gender	−0.235	−0.551	0.081	−0.078	0.144
	**Smoking habits**	−0.361	−0.546	−0.176	−0.204	**<0.001**
	Exposure	0.099	−0.183	0.381	0.037	0.490

^a^ Baseline: Female; ^b^ Baseline: non-smoker; ^c^ Baseline: unexposed. Oxidative damage: tail DNA% Enz—%tail DNA. Bold character indicates statistically significant *p*-values.

**Table 6:** Logistic regression model predicting the likelihood of positivity to Oxidative Damage outcome based on gender, age, smoking habits and exposure.

Independent Variables	Coefficient (β)	Odds Ratio (OR)	95% CI		*p*-Value
			Lower	Upper	
Intercept	−1.514	0.220			<0.001
Female		Ref			
Male	0.064	1.066	0.642	1.772	0.804
Non-smoker		Ref			
Former smoker	0.247	1.280	0.605	2.709	0.519
Smoker	−0.058	0.944	0.514	1.734	0.852
Age	0.012	1.012	0.991	1.033	0.267
Unexposed		Ref			
**Exposed**	0.964	2.623	1.654	4.161	**<0.001**

Bold character indicates statistically significant *p*-values.

**Table 7:** Correlation between Age and DNA damage.

Variables	Total Sample (r, *p*)	Controls (r, *p*)	Exposed (r, *p*)
Age—tail DNA %	−0.020, *p* = 0.698	−0.012, *p* = 0.884	0.006, *p* = 0.932
Age—Oxidative damage (tail DNA%enz-tail DNA%)	0.036, *p* = 0.489	−0.021, *p* = 0.787	0.065, *p* = 0.347
Age—Tail Moment	−0.013, *p* = 0.802	0.032, *p* = 0.687	0.002, *p* = 0.975
Age—Tail Length	−0.009, *p* = 0.861	0.034, *p* = 0.667	0.002, *p* = 0.972

Note. Pearson correlation coefficients (r) are reported along with corresponding *p*-values. Correlations with *p* < 0.05 are considered statistically significant.

**Table 8:** Correlation between BMCyt Assay and fpg-comet assay.

Variables	Total Sample (r, *p*)	Controls (r, *p*)	Exposed (r, *p*)
‰MN—% DNA tail buffer	0.151, ***p* = 0.005**	0.091, *p* = 0.268	0.048, *p* = 0.504
‰MN—% DNA tail enz-%tail DNAbuff	0.088, *p* = 0.100	0.009, *p* = 0.914	0.033, *p* = 0.647
‰MN—Tail Moment	0.177, ***p* = 0.001**	0.038, *p* = 0.643	0.067, *p* = 0.345
‰MN—Tail Length	0.146, ***p* = 0.007**	−0.071, *p* = 0.387	0.061, *p* = 0.392
‰(MN + NB + BE)—% DNA tail buff	0.031, *p* = 0.563	0.070, *p* = 0.394	−0.121, *p* = 0.089
‰(MN + NB + BE)—% DNAtail enz-%DNAtail buf buff	0.063, *p* = 0.240	0.040, *p* = 0.621	−0.001, *p* = 0.991
‰(MN + NB + BE)—Tail Moment	0.087, *p* = 0.103	0.043, *p* = 0.597	−0.043, *p* = 0.550
‰(MN + NB + BE)—Tail Length	0.073, *p* = 0.170	−0.010, *p* = 0.898	−0.027, *p* = 0.702
‰ Cells Condensed Chromatin—%Apoptotic cells	0.026, *p* = 0.598	0.010, *p* = 0.899	0.041, *p* = 0.564
‰Total Anomalies—%tail DNA	0.036, *p* = 0.505	0.080 *p* = 0.333	−0.124 *p* = 0.083

Pearson correlation coefficients (r) are reported along with corresponding *p*-values. Correlations with *p* < 0.05 are considered statistically significant. Bold character indicates statistically significant *p*-values.

## Data Availability

The original contributions presented in this study are included in the article; further inquiries can be directed to the corresponding author.

## Abbreviations

The following abbreviations are used in this manuscript:

AD	Antineoplastic drug
GEM	Gemcitabine
IFO	Ifosfamide
CP	Cyclophosphamide
5-FU	5-Fluorouracil
Pt	Platinum compound
IARC	International Agency for Research on Cancer
NIOSH	National Institute for Occupational Safety and Health
ROS	Reactive Oxygen Species
MN	Micronucleus
NB	Nuclear Bud
BE	Broken Egg
NTP	National Toxicology Program
HPLC	High-Performance Liquid Chromatography
UHPLC	Ultra High-Performance Liquid Chromatography
GC	Gas Chromatography
ICP	Inductively Coupled Plasma
MS	Mass Spectrometry
BMCyt	Buccal Micronucleus Cytome
FPG	Formamido-pyrimidine DNA glycosylase-
ENDOIII	Endonuclease III
HIPEC	Hyperthermic Intraperitoneal Chemotherapy
PIPAC	Pressurized Intraperitoneal Aerosol Chemotherapy
LOD	Limit of Detection
PBS	Phosphate-Buffered Saline
EDTA	Ethylenediaminetetraacetic Acid
DMSO	Dimethyl Sulfoxide
NMA	Normal Melting Agarose
LMA	Low Melting Agarose
TL	Tail Length
TM	Tail Moment
CI	Confidence Interval
Buff	Buffer
Enz	Enzyme
